# Efficacy of Fumaric Acid Esters in the R6/2 and YAC128 Models of Huntington's Disease

**DOI:** 10.1371/journal.pone.0016172

**Published:** 2011-01-31

**Authors:** Gisa Ellrichmann, Elisabeth Petrasch-Parwez, De-Hyung Lee, Christiane Reick, Larissa Arning, Carsten Saft, Ralf Gold, Ralf A. Linker

**Affiliations:** 1 Department of Neurology, St. Josef-Hospital, Ruhr-University Bochum, Bochum, Germany; 2 Department of Neuroanatomy and Molecular Brain Research, Ruhr-University Bochum, Bochum, Germany; 3 Department of Human Genetics, Ruhr-University Bochum, Bochum, Germany; Julius-Maximilians-Universität Würzburg, Germany

## Abstract

Huntington's disease (HD) is an autosomal dominantly inherited progressive neurodegenerative disease. The exact sequel of events finally resulting in neurodegeneration is only partially understood and there is no established protective treatment so far. Some lines of evidence speak for the contribution of oxidative stress to neuronal tissue damage. The fumaric acid ester dimethylfumarate (DMF) is a new disease modifying therapy currently in phase III studies for relapsing-remitting multiple sclerosis. DMF potentially exerts neuroprotective effects via induction of the transcription factor “nuclear factor E2-related factor 2” (Nrf2) and detoxification pathways. Thus, we investigated here the therapeutic efficacy of DMF in R6/2 and YAC128 HD transgenic mice which mimic many aspects of HD and are characterized by an enhanced generation of free radicals in neurons. Treatment with DMF significantly prevented weight loss in R6/2 mice between postnatal days 80–90. At the same time, DMF treatment led to an attenuated motor impairment as measured by the clasping score. Average survival in the DMF group was 100.5 days vs. 94.0 days in the placebo group. In the histological analysis on day 80, DMF treatment resulted in a significant preservation of morphologically intact neurons in the striatum as well as in the motor cortex. DMF treatment resulted in an increased Nrf2 immunoreactivity in neuronal subpopulations, but not in astrocytes. These beneficial effects were corroborated in YAC128 mice which, after one year of DMF treatment, also displayed reduced dyskinesia as well as a preservation of neurons. In conclusion, DMF may exert beneficial effects in mouse models of HD. Given its excellent side effect profile, further studies with DMF as new therapeutic approach in HD and other neurodegenerative diseases are warranted.

## Introduction

Huntington's Disease (HD) is an autosomal dominantly inherited neurodegenerative disorder caused by a trinucleotide CAG repeat expansion ≥36 in the *HD* gene located on chromosome 4. The course of HD is characterized by progressive motor dysfunction, cognitive impairment, affective disorders and personality changes. The most striking neuropathological feature of HD is the progressive atrophy of the striatum and cortex accompanied by neuronal cell loss in these regions [1]. The major histopathological hallmark is the accumulation of intracellular huntingtin (Htt) aggregates, which continuously increase as the disease progresses. The underlying mechanisms of neurodegeneration and Htt aggregation are far less well understood. Here, lack of trophic factors, mitochondrial dysfunction and oxidative stress may play a role [2]. So far, therapeutic options for HD are limited to symptomatic treatment approaches and there is no cure for this devastating disease.

Dimethylfumarate (DMF) is an orally bioavailable fumaric acid ester (FAE) which is metabolized to methyl hydrogen fumarate [3]. Since 50 years, fumaric acid esters are now in clinical use for the treatment of psoriasis [4]. In the recent years, DMF was successfully introduced into the therapy of relapsing-remitting multiple sclerosis (MS). In a first phase IIb study the modified FAE BG00012 was successfully tested and phase III studies are currently ongoing to further explore its therapeutic potential in this indication. While several dermatologic *in vitro* studies point at immunomodulatory effects [5], another primary mode of action of DMF may be the induction of the nuclear factor E2-related factor 2 (Nrf2) pathway which activates detoxifying phase II enzymes [6]. This pathway may thus play a critical role for cellular protection in an environment of oxidative stress and may present an interesting therapeutic target in HD.

Here we explore the potential of DMF in the R6/2 and YAC128 transgenic murine models of HD. The R6/2 and YAC128 mouse strains are well characterized animal models mimicking many histopathological aspects of HD. R6/2 mice trangenetically express the exon 1 of the human *HD* gene with 141–157 CAG repeats [7]. In R6/2 mice, motor symptoms like dyskinesia, ataxia, clasping behaviour, epileptic seizures and spontaneous shivering movements start at the age of about 6 weeks. Continous weight loss leads to death between 11–14 weeks of age. From the age of 9–10 weeks, there is a significant neuronal dysfunction and mice display neuronal atrophy in the striatum [8]. In YAC128 mice, a yeast artificial chromosome (YAC) transgene is employed to express a full-length mutant Htt with about 120 CAG repeats [9], [10]. In YAC128 mice, hypoactivity is first seen at the age of 8 months. Additionally, progressive gait abnormalities, ataxia, hindlimb clasping and a progressive decline in the forced motor function as measured by the rotarod test occur over time [10], [11].

In the models of R6/2 and YAC128 mice, we show that DMF exerts beneficial effects and preserves motor functions as well as intact neurons which may involve activation of the Nrf2 signalling pathway.

## Materials and Methods

### Animal models and treatment procedure

Male R6/2 and YAC128 mice were obtained from the Jackson Laboratories (Bar Harbor, Maine, USA) and bred at the local animal care facilities under standardized conditions. Mice were given food and water at libidum and were weighed daily to obtain weight curves. In the R6/2 strain, stability of the transgene over at least 5 generations of backcrossing was proven by PCR (data not shown). All transgenic mice were heterozygous. All animal experiments were approved by the local authorities for animal experimentation (approval ID: 8.87-50.10.32.08.032, § 8 Protection of Animals Act). For treatment, mice received either DMF 30 mg/kg body weight in 0.08% methocel as carrier solution or the same amount of methocel alone as placebo treatment twice daily via oral gavage. For survival analysis, cohorts of mice were followed over the course of disease with moribund mice sacrificed according to animal protection laws.

### Behavioral analyses

Over the course of the disease, mice were subjected to behavioral analyses every second week starting from week 3. An accelerating rotarod was used to analyse motor coordination and balance (Ugo Basile, Biological Research Apparatus, Varese, Italy). Mice were trained twice on the rotarod with 10 rpm for a maximum of 240 s. During test conditions, the rotarod was accelerated from 4 to 40 rpm with two trials on the same day.

To analyse limb dyskinesia, the clasping behaviour was assessed in a blinded manner as described previously [12], [13]. Briefly, mice were vertically suspended for 45 s approximately 40 cm above ground. Scoring was based on the duration of clasping as follows: clasping >10 s = score 3, 5–10 s = score 2, 1–5 s = score 1, no clasping = score 0.

Gait analysis was performed by footprint analysis as described previously [14]. Analysis included stride-length, hind-base width, front-base width and overlap indicating the distance from left or right front footprint to hind footprint.

Cohorts with a maximal number of 47 R6/2 mice per group (only male mice) and a maximal number of 23 YAC128 mice per group (only male mice) were tested.

### Light- and electronmicroscopical analyses

R6/2 and YAC128 mice were deeply anesthetized with ketamine, transcardially perfused either with 4% paraformaldehyde in phosphate buffered saline (PBS, pH 7.4) for Nissl staining and immunohistochemistry or 2.5% glutaraldehyde and 4% paraformaldehyde in PBS (pH 7.4) for semithin and ultrathin section analyses.

For light microscopy, brains were removed and standardized 2 mm frontal brain slices were prepared using a brain slicer starting 2 mm rostral of the bregma and embedded in paraffin. Three µm paraffin sections were subjected to cresyl violet staining or immunohistochemistry for NeuN (Chemicon MAB377; via Millipore, Schwalbach, Germany; 1∶200) to label neurons, ubiquitin (Chemicon MAB 1510; 1∶1000) or Htt (Chemicon MAB2166; 1∶500) to label Htt aggregates [15], [16], and Mac-3 (BD Pharmingen; Heidelberg, Germany; 1∶200) to label activated microglia/macrophages. Immunohistochemistry for Nrf2 (Santa Cruz Biotechnology C20 sc-722; Santa Cruz, USA; 1∶200) was analyzed by light microscopy or confocal laser scanning microscopy in co-localization studies with GFAP (DAKO, Hamburg, Germany; 1∶1000) to label Nrf2 in astrocytes or NeuN to detect neuronal Nrf2. All histological procedures were essentially performed as described previously [17].

For electron microscopical analysis, removed brains were adjusted in a plexiglass frame following the orientation of Paxinos and Franklin [18], embedded in 2% agarose in PBS and cut into 1.5 mm frontal brain slices with a vibratome cutter. All slices were photodocumented for later orientation, postfixed with 2% osmium tetroxide in PBS for 3 hours and embedded in araldite (Serva, Heidelberg, Germany) as recently described [19]. Blocks with the striatum and motor cortex were prepared for semithin section series, cut with a Leica Ultracut UCT microtome and stained with 1% toluidine (pH 9.3). Ultrathin sections (80 µm) were contrasted with uranyl acetate and lead citrate. Light microscopical pictures were taken by a CP71 camera (Olympus, Hamburg, Germany). Ultrathin sections were viewed in a Philips EM 420 electron microscope documented by the digital system DITABIS (Digital Biomedical Imaging System, Pforzheim, Germany).

### Detection of oxidative chemistry markers in tissue samples

The presence of reactive oxygen species and other oxidants such as ONOO^−^ was visualized on frozen mouse brain sections from R6/2 mice and wild-type controls using dihydroethidium as described previously [20]. After transcardial perfusion with phosphate buffered saline (PBS, pH 7.4), we applied 5 µM hydroethidine (Polysciences, CAS Number 104821-25-2) for 30 minutes. Sections were analyzed by confocal laser scanning microscopy.

### Quantification and statistical analyses

All neuropathological and behavioral analyses were performed completely blinded with respect to treatment allocation. In the striatum or motor cortex of both hemispheres, quantification of neuronal densities was performed on standardized sections comprising an area of 1 mm^2^. On 2 sections per animal, 4 standardized areas were analyzed under 40 fold magnification. Intact neurons were defined by intact shape, regular appearance and intact processes without formation of vacuoles. Data are presented as mean ± SEM. For histological evaluations, statistical analysis was performed by Mann-Whitney U-test. To assess the survival of mice, a Kaplan-Meier analysis with Log rank test was used (all analyses done by Graph Pad Prism 5, San Diego, CA, USA). A probability level of *p<0.05, **p<0.01, ***p<0.001 was considered to be statistically significant for all tests.

## Results

### The symptomatic disease stage in R6/2 mice is associated with an increased formation of oxygen radicals

**Figure 1:**
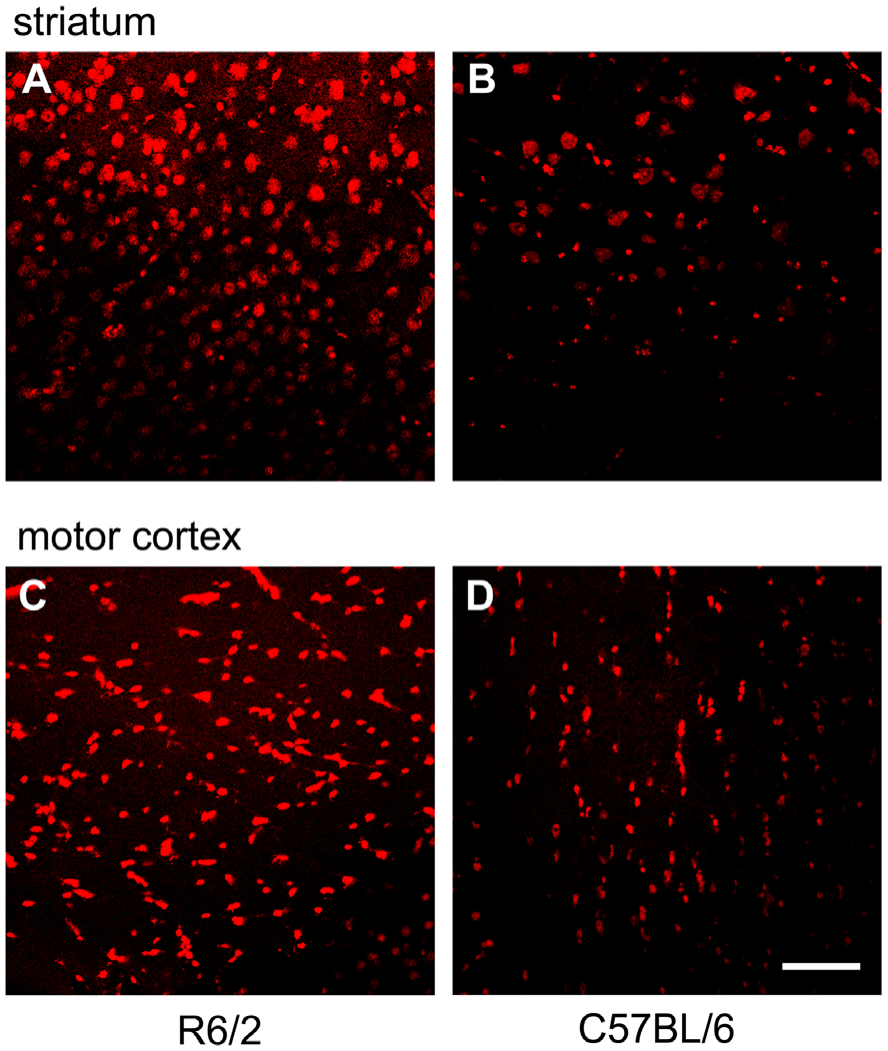
R6/2 mice display higher levels of oxidative stress. (A, B) Representative confocal laser scanning image from the striatum of a 3 months old R6/2 mouse (male, A) after hydroethidine staining in comparison to the striatum of a 4 months old C57BL/6 mouse (B). (C, D) Hydroethidine staining of the motor cortex. The same representative R6/2 mouse (C) and C57BL/6 control mouse (D) as in A,B are shown. The amount of free radicals in neuronal cells is indicated in red. Note the increased immunofluorescence in the R6/2 mouse indicating a higher amount of oxidative stress in both anatomical regions. Bar = 20 µm.

In a first step, we examined the formation of free radicals in R6/2 mice at the age of 3–4 months, i.e. at a time point where mice are also clinically symptomatic and exhibit severe neurodegeneration [21]. Therefore, we stained brain sections with dihydroethidine as a broad-spectrum indicator of oxidative stress. As compared to wild-type mice, R6/2 mice showed a significantly larger amount of reactive oxygen species in neurons, as detected by dihydroethidium staining (Fig. 1).

In summary, neurodegeneration in R6/2 mice is associated with higher levels of oxidative stress in neurons which is well in line with previous studies [22], [23].

### The antioxidant DMF treatment leads to a prolonged survival and preserved body weight in R6/2 mice

**Figure 2:**
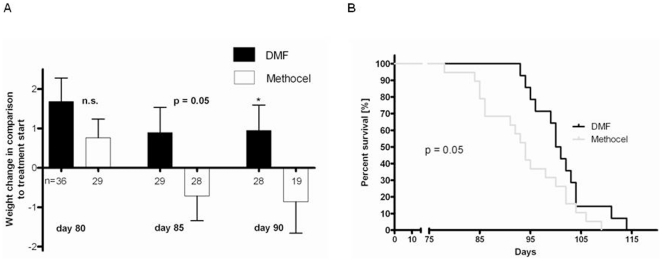
DMF improves survival and prevents weight loss in R6/2 mice. (A) Comparison of body weight changes in DMF treated male R6/2 mice (black bars) and controls treated with carrier solution alone (white bars). Treatment with DMF prevented weight loss in R6/2 mice on postnatal days 85 (p = 0.05) and 90 (p = 0.04). Data are shown as change in body weight (g) on days 80, 85 and 90 to the respective baseline weight at the start of treatment. Numbers of mice per day of analysis are indicated in the graph. (B) Kaplan Meier survival analysis of male R6/2 mice treated with DMF (n = 14, black curve) or methocel (n = 19, grey curve). DMF treatment leads to prolonged survival of R6/2 mice. Confidence interval for mean survival: DMF 97.7–104.6 vs. methocel 91.22–99.6 days.

Since we found a considerable amount of oxidative stress in the R6/2 mouse model of HD, we were interested in the clinical effects of DMF, a compound which has been recently shown to exert neuroprotective effects via the induction of antioxidant pathways [6]. After initiation of treatment with DMF at 30 mg/kg body weight four weeks after birth, R6/2 mice displayed a maximum weight between days 50 to 70 of age and then lost body weight concurrent with the progression of motor deficits. Upon comparison of DMF treated R6/2 mice with controls treated with carrier solution alone, there was a trend to preserved body weight after DMF treatment with a significant preservation of body weight on days 85 and 90 of life (Fig. 2A). Moreover, DMF treated R6/2 mice displayed a 7% increase in life span. In a Kaplan-Meier analysis, the median survival after DMF treatment was 100.5 days in contrast to 94.0 days after oral gavage with vehicle only (Fig. 2B).

In summary, DMF treatment leads to a preserved body weight and prolonged survival in R6/2 mice.

### DMF treatment preserves motor functions in R6/2 mice

**Figure 3:**
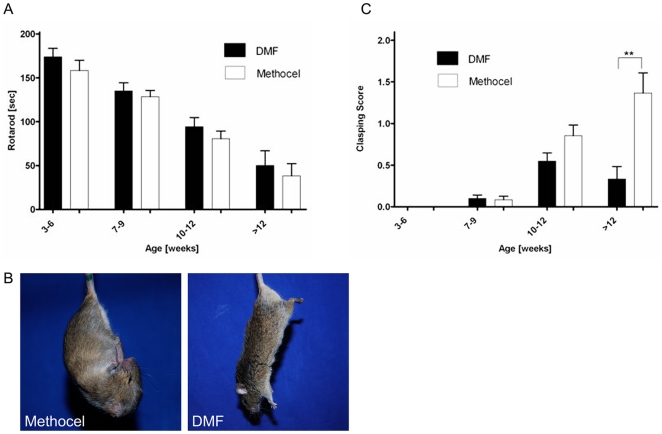
DMF preserves motor functions in R6/2 mice. (A) Rotarod analysis. A cohort of R6/2 mice treated with DMF (n = 46) or methocel (n = 42) is shown. Although DMF treatment (black bars) leads to a trend towards longer times on the accelerating rod, there is no significant difference as compared to methocel treated controls (white bars). (B) Representative images of a R6/2 mouse treated with DMF and a mouse treated with methocel at the age of 12 weeks. Note the clasping of the front- and hindlimbs in the control mouse. (C) Clasping Score. A cohort of R6/2 mice treated with DMF (n = 46, black bars) or methocel (n = 42, white bars) is shown. At the age of 12 weeks, there is a significant difference between both groups and DMF treated mice display a reduction of limb dyskinesia.

In a next step, we correlated the beneficial clinical effects of DMF in the R6/2 mouse model with behavioral tests of motor functions. While motor functions generally decreased over time, analysis of forced motor behaviour in the rotarod test from weeks 10–12 revealed that DMF treatment led to a trend towards increased time on the rotarod (Fig 3A). Furthermore, R6/2 mice were tested weekly for clasping behaviour as a marker of dyskinesia. Up to the age of 9 weeks, there was no difference in clasping scores between DMF treated mice or methocel treated controls (Fig. 3C). At 10–12 weeks of age, matched with the beginning of motor impairment, DMF treatment resulted in a trend towards reduced clasping scores. In mice older than 12 weeks, DMF treatment led to significantly reduced clasping scores as compared to methocel treated controls (Fig. 3B,C). Additionally, gait abnormalities were assessed by analyzing footprint patterns of DMF or sham treated R6/2 mice walking along a narrow tunnel. Analysis of gait width, stride length or overlap did not reveal any significant differences between both groups, but also failed to convincingly show differences over the course of disease between sham-treated R6/2 mice and wild-type controls (data not shown). In summary, DMF treatment resulted in a preservation of motor functions in R6/2 mice.

### DMF treatment preserves the morphology of neurons in the striatum and the motor cortex

**Figure 4:**
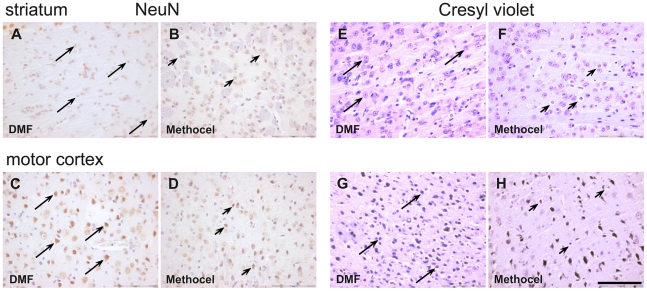
DMF leads to preservation of morphologically intact neurons. Representative images of the striatum (A,B and E,F) or motor cortex (C,D and G,H) from DMF (A,C,E,G) or methocel treated mice (B,D,F,H) on day 80 are shown. Bar = 100 µm. Arrows indicate intact, arrowheads indicate degenerating neurons. (A–D) NeuN staining of the striatum (A,B) and the motor cortex (C,D) in R6/2 mice (day 80). Note the higher number of NeuN immunoreactive neurons after DMF treatment (marked by arrows, arrowheads indicate degenerating striatal neurons with loss of NeuN immunoreactivity). (E–H) Cresyl violet staining of the striatum (E,F) and motorcortex (G,H) in R6/2 mice. Note the higher number of intact neurons after DMF treatment (marked by arrows) and the increased number of shrunken, dystrophic neurons after methocel treatment (marked by arrowheads). False color images depicting cresyl violet staining in red are shown.

Since DMF treatment exerts beneficial effects on survival and motor behaviour in R6/2 mice, we were interested in the histopathological changes under DMF treatment. Numbers of morphologically intact neurons were analyzed after cresyl violet staining or immunohistochemistry for the NeuN antigen on day 80. Morphological criteria were applied to a blinded stereological analysis of neurons in the striatum and motor cortex. In the analysis of both NeuN positive or cresyl violet stained cells, DMF treatment resulted in 1.5–2 fold higher numbers of intact neurons both in the striatum as well as in the motor cortex (Tab. 1 and Fig. 4A–H).

**Table 1:** Quantification of neurons after Cresyl violet and NeuN staining.

Numbers of neurons	Striatum		*p*-value	Motor cortex		*p*-value	n
	DMF	Methocel		DMF	Methocel		
**Cresyl violet** [±SEM]	1075±81	769.9±35	0.0018	1384±65	704.8±81	<0.0001	8/8
**NeuN** [±SEM]	637.8±48	332±30.2	<0.0001	1092±60	640.9±61	<0.0001	8/8

Blinded quantification of neurons after cresyl violet or NeuN staining revealed a significantly higher number of intact neurons in the striatum and in the motor cortex of DMF treated mice.

**Figure 5:**
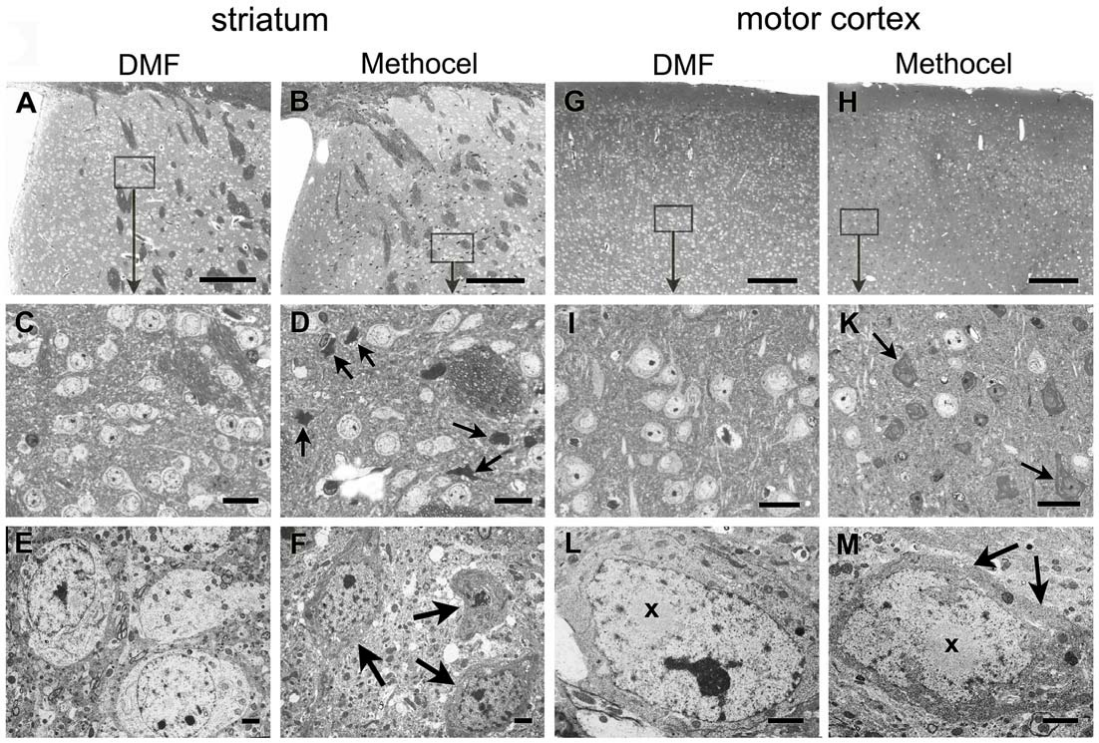
DMF treatment prevents dark cell degeneration in R6/2 mice. (A,B) Overview images of semithin sections show the striatum of DMF (A) and methocel treated (B) R6/2 mice at 80 days of age. Representative images are shown. (C,D) At higher enlargement (for localization see rectangles), the light striatal neurons appear intact in the DMF treated mouse (C) whereas dark cells (arrows) are dispersed among intact neurons in the methocel treated mouse (D). (E,F) Electron microscopy confirms neuronal integrity in DMF treated mice (E) and the condensed and shrunken appearance of severely affected striatal neurons in methocel treated mice (F). (G,H) Semithin overviews display regular cortical morphology of the DMF treated mouse (G), but an irregular appearance in the cortex of representative methocel treated mouse (H). (I,K) At higher enlargements (rectangles) of corresponding layers the DMF treated mouse exhibits intact neurons (I) whereas the methocel treated mouse show multiple dark cells of varying size (arrows) dispersed between single intact neurons (K). (L,M) Electron microscopy confirms the dark cell degeneration with shrunken dark cytoplasm (arrows) and a ruffled nuclear envelope of a respective neuron (M) in contrast to the intact cortical neuron of a DMF treated mouse (L). Both the intact and the dark neuron exhibit a prominent intranuclear round inclusion (x). Bars in A,B,G,H = 100 µm; bars in C,D,I,K = 20 µm; bars in E,F,L,M = 1 µm.

Since it is well known that there is no complete neuronal loss, but still severe neurodegeneration in the striatum and the motor cortex of R6/2 mice, the light microscopical data were corroborated by semithin- and ultrasection analysis (Fig. 5). The integrity of striatal and cortical neurons was relatively preserved in DMF treated R6/2 mice (Fig. 5A,C,E,G,I,L) when compared with the corresponding areas in the methocel treated controls (Fig. 5B,D,F,H,K,M). In semithin sections of the striatum (Fig. 5A,C) and of the motor cortex (Fig. 5G,I) of DMF treated mice, most neurons appeared intact in the striatum and motor cortex (Fig. 5A,C,G,I). In methocel treated mice, dark cells were dispersed among intact neurons in the striatum and the motor cortex (Fig. 5B,D,H,K). Electron microscopical analysis confirmed the presence of degenerating neurons with darkened and shrunken appearance in the striatum (Fig. 5F) and in the motor cortex (Fig. 5M) when compared with intact light appearing neurons in the respective areas of DMF treated mice (Fig. 5E,L). Nuclear aggregates were present within intact neurons (Fig. 5L) and also in neurons undergoing dark cell degeneration (Fig. 5M).

In summary, DMF treatment led to preservation of intact neurons in the striatum and the motor cortex.

### DMF treatment in R6/2 mice targets the Nrf2 pathway

After having established that DMF exerts beneficial effects on neuronal integrity in R6/2 mice, we next analyzed the mechanisms of DMF action. First, we investigated the effects of DMF on the formation of Htt aggregates. To this end, we performed immunohistochemistry for ubiquitin which reliably detects Htt inclusions in R6/2 mice [15]. DMF treatment did not affect the formation of Htt aggregates as measured by Htt or ubiquitin immunoreactivity (Tab. 2). Similarly, staining for Mac-3 as a marker of activated phagocytes did not disclose a major bystander immune reaction which could be modulated by DMF treatment (data not shown).

**Table 2:** DMF does not influence aggregate formation in R6/2 mice.

Positive neurons	Striatum		*p*-value	Motor cortex		*p*-value	n
	DMF	Methocel		DMF	Methocel		
**Huntingtin numbers**	618.2±54	613.2±28	n.s.	541.0±5	494.7±69	n.s.	8/8
**Ubiquitin %** [±SEM]	52.88±4	47.59±3	n.s.	n.d.	n.d.	-----	8/8

Blinded quantification of the absolute number of huntingtin positive profiles in the striatum or the motor cortex and the percentage of ubiquitin immunoreactive neurons in the striatum did not reveal a significant difference between the DMF and methocel treated groups.

**Figure 6:**
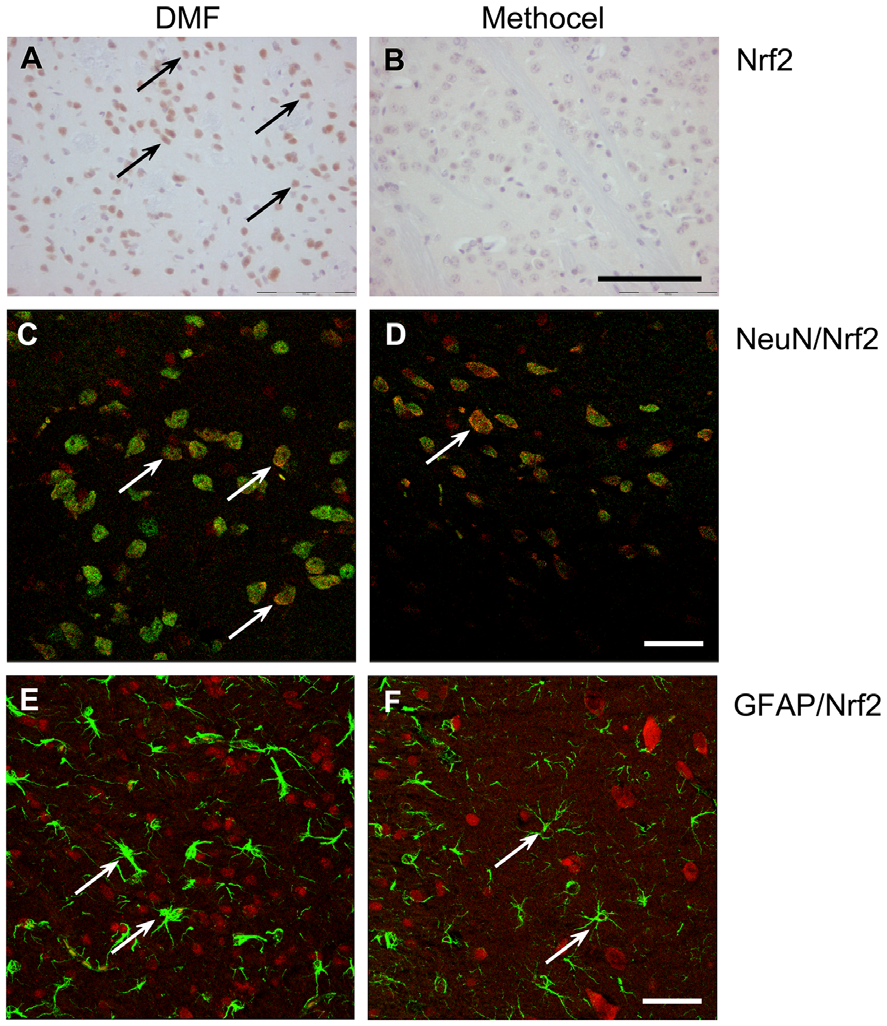
Increased Nrf2 immunoreactivity after DMF treatment. Representative images of the striatum from DMF (A, C, E) or methocel treated mice (B, D, F) on day 80 are shown. (A,B) In contrast to methocel treated R6/2 mice, there is an increased number of Nrf2 positive cells after DMF treatment (Nrf2 immunopositive cells are marked by arrows). Bar = 100 µm. (C,D) Confocal laser scanning microscopy images after NeuN/Nrf2 double staining. In contrast to vehicle treated mice (D), a higher number of NeuN/Nrf2 double positive cells are observed in the striatum in DMF treated R6/2 mice (C). Arrows mark NeuN/Nrf2 double labelled cells. Bar = 20 µm. (E,F) Confocal laser scanning microscopy images after GFAP/Nrf2 double labelling. In the striatum, significant numbers of Nrf2 immunopositive astrocytes were neither identified after DMF treatment (E), nor in control mice (F). Arrows indicate Nrf2 negative astrocytes. Bar = 20 µm; images represent a Z-stack of 10 µm.

DMF is a known inducer of phase 2 detoxifying enzymes and both DMF and its primary metabolite monomethlyfumarate (MMF) are thiol-reactive electrophiles [24], [25], [26]. This combination of properties suggests that the DMF may activate the Nrf2 transcriptional pathway known to mediate induction of phase 2 genes by SH-reactive electrophiles and play a major role in cell and tissue defense against oxidative stress. Immunohistochemistry for Nrf2 revealed a higher number of Nrf2 positive cells in the striatum of DMF treated mice (Fig. 6A,B and Tab. 3). We next analyzed the effects of DMF on Nrf2 expression in different cell types of the striatum and motor cortex by double label immunfluorescence and laser scanning confocal microscopy. Double staining of Nrf2 with the neuronal marker NeuN revealed an increased Nrf2 immunoreactivity after DMF treatment in different neuronal subpopulations of the brain, especially in the striatum and in the motor cortex (Fig. 6 C,D). After double staining of Nrf2 with the astroglial marker GFAP, double labelled cell were neither identified in DMF nor in methocel treated mice (Fig. 6 E, F).

**Table 3:** Quantification of Nrf2 positive cells in the striatum.

Positive neurons	Striatum		*p*-value	n
	DMF	Methocel		
**Nrf2** [numbers ± SEM]	579.1±71	345.6±25	0.0282	7/5

Blinded quantification of Nrf2 positive cells in the striatum revealed a significantly higher number of Nrf2 positive cells in DMF treated mice.

In summary, DMF treatment in R6/2 mice led to an increase in Nrf2 staining in neuronal subpopulations which are relevant for motor functions.

### 3.5 DMF exerts positive effects in the YAC128 model of HD

**Figure 7:**
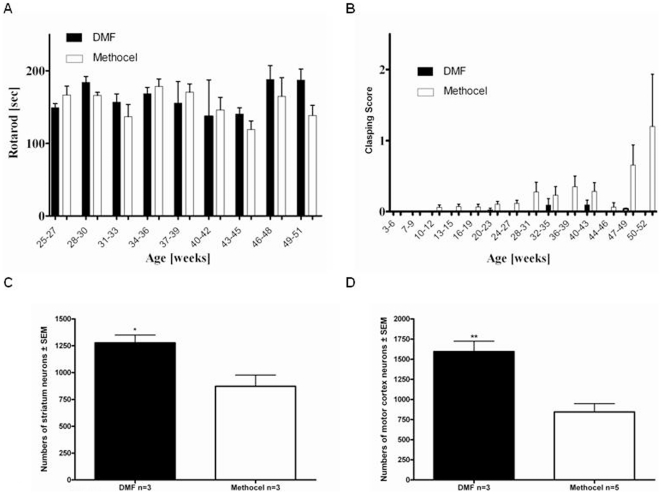
DMF preserves motor functions in YAC128 mice. (A) Rotarod analysis. A cohort of YAC128 mice treated with DMF (n = 23, black bars) or methocel (n = 22, white bars) is shown. Although DMF treatment leads to a trend towards longer times on the accelerating rod, there is no significant difference between both groups. (B) Clasping Score. A cohort of YAC128 mice treated with DMF (n = 23) or methocel (n = 22) is shown. In comparison to control mice (white bars), there is a trend towards reduced clasping behaviour in the DMF treated group (black bars) from the age of 47 weeks. (C) Blinded quantification of neuronal densities in the striatum after creysl violet staining on day 365 (n = 3 per group). There is a significant preservation of neuronal numbers after DMF treatment (black bar) as compared to methocel treatment (white bar, p = 0.03). (D) Blinded quantification of neuronal densities in the motor cortex after creysl violet staining on day 365 (n = 3/5 per group). There is a significant preservation of neuronal numbers after DMF treatment (black bar) as compared to methocel treatment (white bar, p = 0.004).

Finally, the beneficial effects of DMF in the R6/2 mice were corroborated in the YAC128 mouse as another well established mouse model of HD. Analysing the forced motor behaviour on a rotarod confirmed a trend towards preserved motor functions after DMF treatment of YAC128 mice older than 22 weeks (Fig. 7A). While there were no differences in body weight when analysing mice up to a life span of one year (data not shown), DMF treatment in YAC128 mice led to a reduction of limb dyskinesia as measured by the clasping score (Fig. 7B). These beneficial effects on the motor behavior were also seen in a histopathological analysis on day 365. Blinded quantification of intact neurons in the striatum and motor cortex after cresyl violet staining revealed a preservation of neuronal number after DMF treatment (Fig. 7C,D).

In summary, the beneficial effects of DMF in R6/2 mice were also observed in YAC128 mice as another transgenic mouse model of HD.

## Discussion

We show that DMF treatment exerts beneficial effects on survival time and motor functions in the R6/2 and YAC128 models of HD. The clinical efficacy of DMF in the R6/2 model was corroborated by preservation of intact neurons and less pronounced dark cell degeneration in the striatum and motor cortex as well as an up-regulation of the transcription factor Nrf2 in striatal neurons.

As expected, R6/2 mice display a higher amount of free radicals in the CNS indicating an increase in oxidative stress which may be linked to neurodegeneration. Indeed, oxidative stress is known to be a common underlying mechanism in the pathogenesis of many neurodegenerative diseases causing damage to multiple cellular components such as DNA, proteins and lipids [27], [28]. Here, fumaric acid esters which activate antioxidant response pathways may provide neuroprotective effects [6]. Several studies revealed that DMF is a potent inducer of phase 2 detoxifying enzymes [26] and both DMF and its primary metabolite MMF are thiol-reactive electrophiles [25]. These characteristics imply that fumarates may activate the Nrf2 transcriptional pathway which is known to mediate induction of phase 2 genes by SH-reactive electrophiles and to play a major role in cell and tissue defense against oxidative stress [29]. Indeed, several *in vitro* studies described protective effects of DMF on neurons [30], [31]. In cell culture, MMF protected cultured neurons and astrocytes from oxidative stress induced by H_2_O_2_ [6]. Other studies describing deprivation of glutathione or toxicity of DMF and MMF in glial cultures used much higher, non-physiological concentrations of both compounds [32], [33]. Very recent studies using Nrf2 deficient mice in a chronic model of multiple sclerosis provide direct evidence for DMF mediated neuroprotective effect via Nrf2 mediated regulation of the oxidative stress response [6]. Fumarates directly modify the inhibitor of Nrf2, Keap-1, which leads to transfer of free Nrf2 into the nucleus and activation of anti-oxidative response elements. A result of the activated Nrf2 pathway is cellular detoxification, normalization of the energy metabolism and repair and/or degradation of damaged proteins.

The Nrf2-mediated oxidative stress response was already previously linked to protection of the CNS in a variety of pathological conditions [34], [35], [36], [37]. In particular, the importance of this pathway was recently implicated in the pathogenesis of HD [38]. Furthermore, *ex vivo* studies and analyses of neurodegenerative models for motor neuron disorders and Parkinson's disease disclosed that Nrf2 mediated neuroprotection may not only be directly mediated, but in some models critically involves effects via astrocytes [39], [40], [41], [42].

In addition to its neuroprotective capacity, DMF may also possess immunomodulatory effects which were at least proposed in several dermatologic analyses *in vitro* [43], [44], [45], [46], [47]. In the R6/2 model, we did not observe a significant bystander immune reactivity in the CNS which could be modulated by DMF. We thus propose that DMF treatment in HD models primarily targets neurons. This concept is in line with the observation that DMF metabolites reach significant concentrations in the CNS [6]. To further explore the neuroprotective potential of DMF, additional studies in other models of HD and neurodegenerative diseases are warranted.

The efficacy of DMF as an inducer of the oxidative stress response in models of HD underlines the importance of free radicals in the pathophysiology of HD. Indeed, mitochondrial dysfunction, oxidative stress and energy depletion have long been implicated in the pathogenesis of HD and stimulated several therapeutic approaches. In particular, the treatment of R6/2 transgenic mice with dichloracetate which increases the activity of the pyruvate dehydrogenase complex revealed positive effects. Further studies employing antioxidants like α-lipoic acid [48] observed beneficial effects and a significantly enhanced survival of R6/2 mice. Similar results were achieved by administration of co-enzyme Q_10_ [49]. While these effects may be based on the improvement of mitochondrial function or the decrease of oxidative damage, it seems conceivable that different disease stages are governed by different prevailing pathomechanisms. This concept could explain the decreasing efficacy of DMF at the later disease stages in R6/2 mice.

In R6/2 mice, DMF leads to a preservation of intact neurons as revealed by cresyl violet staining and immunohistochemistry for the NeuN antigen. These light microscopy results were corroborated by semi- and ultrathin section analyses revealing a less pronounced dark cell degeneration after DMF treatment. While several reports point at a significant neuronal loss and striatal atrophy in R6/2 mice [7], [8], [50] or YAC128 HD transgenic mice [9], [10], other studies only describe functional changes of neuronal subpopulations in these models [51], [52]. This concept was further refined by the characterization of dark cell degeneration in striatal and cortical neurons [8], [53], [54], [55]. In our study, we therefore used several histological approaches to describe a relative preservation of morphologically intact neurons under DMF treatment as compared to sham treated controls. Yet, the study design does not allow conclusions on a putative overall loss of striatal or motor cortex neuron numbers in R6/2 or YAC128 mice.

Many therapeutic approaches were tested in rodent models of HD, including inhibition of histone deacetylation and methylation [56], [57], congo red [58], [59], disaccharides [60], tiagabine [61], transglutaminase inhibitors [62], [63], caspase inhibitors [64] or also tetracyclins [65], [66], [67]. Yet, many of these approaches were later refuted in clinical trials or could not be reproduced in follow-up studies as recently shown e.g. for minocyline or co-enzyme Q [66], [68]. This may in part be explained by the low number of animals used, environmental factors or husbandry [69]. These ambiguous results highlight the importance of a standardized testing which was also implemented in the present study design [69]. Using large cohorts as well as standardized therapy, husbandry and testing batteries, we show that DMF exerts beneficial effects in the two best characterized mouse models of HD. While DMF does not influence the pathogensis of HD, it exerts significant protective effects on the affected tissue. Given its excellent side effect profile, further studies with DMF as a potential neuroprotective approach in HD are warranted. Such trials may also include combination therapy approaches to target different pathomechanisms of HD which are possibly stage-specific.

## References

[1] Vonsattel JP, DiFiglia M (1998). Huntington disease.. J Neuropathol Exp Neurol.

[2] Kumar P, Kalonia H, Kuman A (2010). Huntington's disease: pathogenesis to animal models.. Pharmacol Rep.

[3] Mrowietz U, Christophers E, Altmeyer P (1999). Treatment of severe psoriasis with fumaric acid esters: scientific background and guidelines for therapeutic use. The German Fumaric Ester Consensus Conference.. Br J Dermatol.

[4] Schweckendiek W (1959). Treatment of psoriasis vulgaris.. Med Monatsschr.

[5] Linker RA, Lee DH, Stangel M, Gold R (2008). Fumarates for the treatment of multiple sclerosis, potential mechanisms of actions and clinical studies.. Expert Rev Neurother.

[6] Linker RA, Lee DH, Ryan S, van Dam AM, Conrad R Fumaric acid esters exert neuroprotective effects in neuroinflammation via activation of the Nrf2 antioxidant pathway.. Brain.

[7] Mangiarini L, Sathasivam K, Seller M, Cozens B, Harper A (1996). Exon 1 of the HD gene with an expanded CAG repeat is sufficient to cause a progressive neurological phenotype in transgenic mice.. Cell.

[8] Stack EC, Kubilius JK, Smith K, Cormier K, Del Signore SJ (2005). Chronology of behavioral symptoms and neuropathological sequela in R6/2 Huntington's disease transgenic mice.. J Comp Neurol.

[9] Hodgson JG, Agopyan N, Gutekunst CA, Leavitt BR, LePiane F (1999). A YAC mouse model for Huntington's disease with full-length mutant huntingtin, cytoplasmatic toxicity, and selective striatal neurodegeneration.. Neuron.

[10] Slow EJ, van Raamsdonk J, Rogers D, Coleman SH, Graham RK (2003). Selective striatal neuronal loss in a YAC128 mouse model of Huntington disease.. Hum Mol Genet.

[11] van Raamsdonk JM, Pearson J, Slow EJ, Hossain SM, Leavitt BR (2005). Cognitive dysfunction precedes neuropathology and motor abnormalities in the YAC128 mouse model of Huntington's disease.. J Neurosci.

[12] Pang TY, Stam NC, Nithianantharajah J, Howard ML, Hannan AJ (2006). Differential effects of voluntary physical exercise on behavioral and brain-derived neurotrophic factor expression deficits in Huntington's disease transgenic mice.. Neuroscience.

[13] van Dellen A, Blakemore C, Deacon R, York D, Hannan AJ (2000). Delaying the onset of Huntington's in mice.. Nature.

[14] Clarke KA, Still J (1999). Gait Analysis in the Mouse.. Physiol and Behav.

[15] Davies SW, Turmaine M, Cozens B, DiFiglia M, Sharp AH (1997). Formation of neuronal intranuclear inclusions underlies the neurological dysfunction in mice transgenic for the HD mutation.. Cell.

[16] Li H, Li SH, Yu ZX, Shelbourne P, Li XJ (2001). Huntingtin aggregate-associated axonal degeneration is an early pathological event in Huntington's disease mice.. J Neurol.

[17] Linker RA, Mäurer M, Gaupp S, Martini R, Holtmann B (2002). CNTF is a major protective factor in demyelinating CNS disease: a neurotrophic cytokine as modulator in neuroinflammation.. Nat Med.

[18] Paxinos G, Franklin KB (2007). The mouse brain in stereotaxic coordinates.

[19] Petrasch-Parwez E, Nguyen HP, Löbbecke-Schumacher M, Habbes HW, Wieczorek S (2007). Cellular and subcellular localization of Huntingtin [corrected] aggregates in the brain of a rat transgenic for Huntington disease.. J Comp Neurol.

[20] Kleinschnitz C, Grund H, Wingler K, Armitage ME, Jones E (2010). Post-stroke inhibition of induced NADPH oxidase type 4 prevents oxidative stress and neurodegeneration.. PloS Biol.

[21] Carter RJ, Lione LA, Humby T, Mangiarini L, Mahal A (1999). Characterization of Progressive Motor Deficits in Mice Transgenic for the Human Huntington's Disease Mutation.. J Neurosci.

[22] Browne SE, Bowling AC, MacGarvey U, Baik MJ, Berger SC (1997). Oxidative damage and metabolic dysfunction in Huntington's disease: selective vulnerability of the basal ganglia.. Ann Neurol.

[23] Bogdanov MB, Andreassen OA, Dedeoglu A, Ferrante RJ, Beal MF (2001). Increased oxidative damage to DNA in a transgenic mouse model of Huntington's disease.. J Neurochem.

[24] Begleiter A, Sivananthan K, Curphey TJ, Bird RP (2003). Induction of NAD(P)H quinone: oxidoreductase1 inhibits carcinogen-induced aberrant crypt foci in colons of Sprague-Dawley rats.. Cancer Epidemol Biomarkers Prev.

[25] Schmidt TJ, Ak M, Mrowietz U (2007). Reactivity of dimethyl fumarate and methylhydrogen fumarate towards glutathione and N-acetyl-L-cysteine-preparation of S-substituted thiosuccinic acid esters.. Bioorg Med Chem.

[26] Wierinckx A, Breve J, Mercier D, Schultzberg M, Drukarch B (2005). Detoxication enzyme inducers modify cytokine production in rat mixed glial cells.. J Neuroimmunol.

[27] Fernández-Checa JC, Fernández A, Morales A, Marí M, García-Ruiz C (2010). Oxidative stress and altered mitochondrial function in neurodegenerative diseases: lessons from mouse models.. CNS Neurol Disord Drug Targets.

[28] Ang ET, Tai YK, Lo SQ, Seet R, Soong TW (2010). Neurodegenerative diseases: exercising toward neurogenesis and neurodegeneration.. Front Aging Neurosci.

[29] Jung KA, Kwalk MK (2010). The Nrf2 system as a potantial target for the development of indirect antioxidants.. Molecules.

[30] Duffy S, So A, Murphy TH (1998). Activation of endogenous antioxidant defenses in neuronal cells prevents free radical-mediated damage.. J Neurochem.

[31] Su JY, Duffy S, Murphy TH (1999). Reduction of H2O2 evoked, intracellular caldium increases in the rat N18-RE-105 neuronal cell line by pretreatment with an electrophilic antioxidant inducer.. Neurosci Lett.

[32] Moharregh-Khiabani D, Blank A, Skripuletz T, Miller E, Kotsiari A (2010). Effects of fumaric acids on cuprizone induced central nervous system de- and remyelination in the mouse.. PLoS One.

[33] Thiessen A, Schmidt MM, Dringen R (2010). Fumaric acid dialkyl esters deprive cultured rat oligodendroglial cells of glutathione and upregulate the expression of heme oxygenase 1.. Neurosci Lett.

[34] Calkins MJ, Jakel RJ, Johnson DA, Chan K, Kan YW (2005). Protection from mitochondrial complex II inhibition in vitro and in vivo by Nrf2-mediated transcription.. Proc Natl Acad Sci.

[35] Lee JM, Shih AY, Murphy TH, Johnson JA (2003). NF-E2-related factor-2 mediates neuroprotection against mitochondrial complex I inhibitors and increased concentrations of intracellular calcium in primary cortical neurons.. J Biol Chem.

[36] Satoh T, Okamoto SI, Cui J, Watanabe Y, Furunta K (2006). Activation of the Keap1/Nrf2 pathway for neuroprotection by electrophilic [correction of electrophilic] phase II inducers.. Proc Natl Acad Sci U S A.

[37] Shih AY, Imbeault S, Barakauskas V, Erb H, Jiang L (2005). Induction of the Nrf2-driven antioxidant response confers neuroprotection during mitochondrial stress in vivo.. J Biol Chem.

[38] Stack C, Ho D, Wille E, Calingasan NY, Williams C (2010). Triterpenoids CDDO-ethyl amide and CDDO-trifluoroethyl amide improve the behavioral phenotype and brain pathology in a transgenic mouse model of Huntington's disease.. Free Radic Biol Med.

[39] Chen PC, Vargas MR, Pani AK, Smeyne RJ, Johnson DA (2009). Nrf2-mediated neuroprotection in the MPTP mouse model of Parkinson's disease: Critical role for the astrocyte.. Proc Natl Acad Sci U S A.

[40] Kraft AD, Johnson DA, Johnson JA (2004). Nuclear factor E2-related factor 2-dependent antioxidant response element activation by tert-buthylhydroquinone and sulforaphane occuring preferentially in astrocytes conditions neurons against oxidative insult.. J Neurosci.

[41] Shih AY, Johnson DA, Wong G, Kraft AD, Jiang L (2003). Coordinate regulation of glutathione biosynthesis and release by Nrf2-expressing glia potently protects neurons from oxidative stress.. J Neurosci.

[42] Vargas MR, Johnson DA, Sirkis DW, Messing A, Johnson JA (2008). Nrf2 activation in astrocytes protects against neurodegeneration in mouse models of familial amyotrophic lateral sclerosis.. J Neurosci.

[43] De Jong R, Bezemer AC, Zomerdijk TP, Pouw-Kraan T, Ottenhoff TH (1996). Selective stimulation of T helper 2 cytokine responses by the anti-psoriasis agent monomethylfumarate.. Eur J Immunol.

[44] Loewe R, Holnthoner W, Groger M (2002). Dimethylfumarate inhibits TNF-induced nuclear entry of NF-kappa B/p65 in human endothelial cells.. J Immunol.

[45] Ockenfels HM, Schultewolter T, Ockenfels G, Funk R, Goos M (1998). The antipsoriatic agent dimethylfumarate immunomodulates T-cell cytokine secretion and inhibits cytokines of the psoriatic cytokine network.. Br J Dermatol.

[46] Sebok B, Bonnekoh B, Vetter R, Schneider I, Gollnick H (1998). The antipsoriatic dimethyl-fumarate suppresses interferon-gamma-induced ICAM-d1 and HLA-DR expression on hyperproliferative keratinocytes. Quantification by a culture plate-directed APAAP-ELISA technique.. Eur J Dermatol.

[47] Vandermeeren M, Janssens S, Borgers M, Geysen J (1997). Dimethylfumarate is an inhibitor of cytokine-induced E-selectin, VCAM-1, and ICAM-1 expression in human endothelial cells.. Biochem Biophys Res Commun.

[48] Clifford JJ, Drago J, Natoli AL, Wong JYF, Kinsella A (2002). Essential fatty acids given from conception prevent topographies of motor deficits in a transgenic model of Huntington's disease.. Neuroscience.

[49] Smith KM, Matson S, Matson WR, Cormier K, Del Signore SJ (2006). Dose ranging and efficacy study of high-dose coenzyme Q10 formulations in Huntington's disease mice.. Biochem Biophys Acta.

[50] Keene CD, Rodrigues CM, Eich T, Chhabra MS, Steer CJ (2002). Tauroursodeoxycholic acid, a bile acid, is neuroprotective in a transgenic animal model of Huntington's disease.. Proc Natl Acad Sci U S A.

[51] Gil JM, Rego C (2009). The R6 lines of transgenic mice: A model for screening new therapies for Huntington's disease.. Brain Res Rev.

[52] Menalled LB, Sison JD, Wu Y, Olivieri M, Li XJ (2002). Early motor dysfunction and striosomal distribution of huntingtin microaggregates in Huntington's disease knock-in mice.. J Neurosci.

[53] Iannicola C, Moreno S, Oliverio S, Nardacci R, Ciofi-Luzzatto A (2000). Early alterations in gene expression and cell morphology in a mouse model of Huntington's disease.. J Neurochem.

[54] Turmaine M, Raza A, Mahal A, Mangiarini L, Bates GP (2000). Nonapoptotic neurodegeneration in a transgenic mouse model of Huntington's disease.. Proc Natl Acad Sci U S A.

[55] Yu ZX, Li SH, Evans J, Pillarisetti A, Li H (2003). Mutant huntingtin causes context-dependent neurodegeneration in mice with Huntington's disease.. J Neurosci.

[56] Ferrante RJ, Kubilus JK, Lee J, Ryu H, Beesen A (2003). Histone deacetylase inhibition by sodium butyrate chemotherapy ameliorates the neurodegenerative phenotype in Huntington's disease mice.. J Neurosci.

[57] Hockly E, Richon VM, Woodman B, Smith DL, Zhou X (2003). Suberoylanilide hydroxamic acid, a histone deacetylase inhibitor, ameliorates motor deficits in a mouse model af Huntington's disease.. Proc Natl Acad Sci U S A.

[58] Heiser V, Scherzinger E, Boeddrich A, Nordhoff E, Lurz R (2000). Inhibition of huntingtin fibrillogenesis by specific antibodies and small molecules: implication for Huntington's disease therapy.. Proc Natl Acad Sci U S A.

[59] Klunk WE, Pettegrew JW, Abraham DJ (1989). Quantitative evaluation of Congo red binding to amyloid-like proteins with a beta-pleated sheet conformation.. J Histochem Cytochem.

[60] Tanaka M, Machida Y, Niu S, Ikeda T, Jana NR (2004). Trehalose alleviates polyglutamine-mediated pathology in a mouse model of Huntington's disease.. Nat Med.

[61] Masuda N, Peng Q, Li Q, Jiang M, Liang Y (2004). Tiagabine is neuroprotective in the N171-82Q and R6/2 mouse models of Huntington's disease.. Neurobiol Dis.

[62] Dedeoglu A, Kubilus JK, Jeitner TM, Matson SA, Bogdanov M (2002). Therapeutic effects of cystamine in a murine model of Huntington's disease.. J Neurosci.

[63] Karpuj MV, Becher MW, Springer JE, Chabas D, Youssef S (2002). Prolonged survival and decreased abnormal movements in transgenic model of Huntington disease, with administration of the transglutaminase inhibitor cystamine.. Nat Med.

[64] Wellington CL, Ellerby LM, Hackam AS, Margoli RL, Trifiro MA (1998). Caspase cleavage of gene products associated with triplet expansion disorders generates truncated fragments containig the polyglutamine tract.. J Biol Chem.

[65] Chen M, Ona VO, Li M, Ferrante RJ, Fink KB (2000). Minocycline inhibitis caspase-1 and caspase-3 expression and delays mortality in a transgenic mouse model of Huntington's disease.. Nat Med.

[66] Smith DL, Woodman B, Mahal A, Sathasivam K, Ghazi-Noori S (2003). Minocycline and doxycycline are not beneficial in a model of Huntington's disease.. Ann Neurol.

[67] Wang X, Zhu S, Drozda M, Zhang W, Stavrovskaya IG (2003). Minocycline inhibits caspase-independent and –dependent mitochondrial cell death pathways in models of Huntington's disease.. Proc Natl Acad Sci U S A.

[68] Menalled LB, Patry M, Ragland N, Lowden PAS, Goodmann J (2010). Comprehensive behavioral testing in the R6/2 mouse model of Huntington's disease shows no benefit from CoQ10 or Minocycline.. Plos One.

[69] Menalled LB, El-Khodor BF, Patry M, Suarez-Farinas M, Orenstein SJ (2009). Systematic behavioral evaluation of Huntington's disease transgenic and knock-in mouse models.. Neurobiol Dis.

